# The Peculiarities of Large Intron Splicing in Animals

**DOI:** 10.1371/journal.pone.0007853

**Published:** 2009-11-16

**Authors:** Samuel Shepard, Mark McCreary, Alexei Fedorov

**Affiliations:** 1 Department of Medicine, University of Toledo, Toledo, Ohio, United States of America; 2 Rochester Institute of Technology, Rochester, New York, United States of America; University of Western Cape, South Africa

## Abstract

In mammals a considerable 92% of genes contain introns, with hundreds and hundreds of these introns reaching the incredible size of over 50,000 nucleotides. These “large introns” must be spliced out of the pre-mRNA in a timely fashion, which involves bringing together distant 5′ and 3′ acceptor and donor splice sites. In invertebrates, especially Drosophila, it has been shown that larger introns can be spliced efficiently through a process known as recursive splicing—a consecutive splicing from the 5′-end at a series of combined donor-acceptor splice sites called RP-sites. Using a computational analysis of the genomic sequences, we show that vertebrates lack the proper enrichment of RP-sites in their large introns, and, therefore, require some other method to aid splicing. We analyzed over 15,000 non-redundant, large introns from six mammals, 1,600 from chicken and zebrafish, and 560 non-redundant large introns from five invertebrates. Our bioinformatic investigation demonstrates that, unlike the studied invertebrates, the studied vertebrate genomes contain consistently abundant amounts of direct and complementary strand interspersed repetitive elements (mainly SINEs and LINEs) that may form stems with each other in large introns. This examination showed that predicted stems are indeed abundant and stable in the large introns of mammals. We hypothesize that such stems with long loops within large introns allow intron splice sites to find each other more quickly by folding the intronic RNA upon itself at smaller intervals and, thus, reducing the distance between donor and acceptor sites.

## Introduction

Introns are found ubiquitously in eukaryotic genomes and yet their role is still poorly understood and underappreciated. A range of recent studies have suggested that introns may have even existed in what some regard to be primordial eukaryotes [1]–[4] or even earlier [5]–[6]. Different aspects of the evolution of introns have been well reviewed by [7]–[9].

About 92% of mammalian genes have exon/intron structures while only 8% of genes are intron-free. The average segmented gene of these species contain between 8 and 9 introns. The total length of introns represents 35–40% of the euchromatic portion of mammalian genomes. Many introns are extremely long. For example, there are over 3000 human introns larger than 50 kb, 1,234 longer than 100 kb, 299 longer than 200 kb, and 9 longer than 500 kb [10]. The enormous size of introns in mammals creates several drawbacks. First, large introns waste considerable amounts of energy during transcription that is “unwisely” spent on polymerizing the extra-long intronic segments of pre-mRNA molecules. Second, large introns delay obtaining protein products. Third, large introns allow for more potential errors in intron splicing since large introns contain numerous false splice sites (the so-called “pseudo-exons” [11]). It follows that some benefit must therefore be associated with introns to compensate for these costly disadvantages. Different constructive roles for introns are described in [10].

In particular, we concentrate on the problems that large introns (>50 kb) pose to their host genes. During the initial steps of splicing, the 5′-terminus of an intron is brought close to the downstream 3′-terminus by the spliceosome RNA-protein complex. This spatial formation allows the phosphodiester bond at the donor splice site to be attacked by the 2′-OH group of an adenosine residue from a so-called “branch point” located just in front of the acceptor splice site (on average, about 30 bases upstream). The larger the intron, the more remote its ends are from each other. At first approximation, the difficulty of bringing an intron's termini together in our three-dimensional world is proportional to the cube of the intron's length. Therefore, for a large 100 kb intron, it is one million times harder to bring its ends together than for a medium-sized intron of 1 kb in length. In fact, a stretched 100 kb RNA molecule spreads out over a distance of 30 microns, which is larger than the size of mammalian nucleus (about 5 microns). Moreover, splicing of large introns already takes extra time because there are so many bases to transcribe in the first place. Indeed, it takes about 45 minutes for RNA polymerase II to transcribe a 100 kb gene region. Thus, there is a fundamental question: How do large introns manage to splice at all?

Hatton *et al.* [12] as well as Burnette *et al.* [13] showed that Drosophila large introns undergo a process called *recursive splicing*; that is, several pieces of the intron are spliced consecutively starting from the 5′-end. According to Burnette and colleagues, recursive splicing is achieved using a combined donor-acceptor splice site called the “ratcheting point” (or RP-sites). These RP-sites have a consensus of *(y)_n_ncag|gtaagt*, where the splice junction is shown as vertical bar. The consensus sequences of the donor and acceptor splice sites are *AG|gtaagt* and *(y)_n_ncag|GT* respectively (exon terminal sequences are shown in upper case and intron sequences are given in lower case). It is possible therefore that RP-sites may perform both functions–serving as either the 3′- or 5′- splicing junction—in order to facilitate recursive splicing. In 1998, Hatton *et al.* [12] described the existence of recursive splicing in fruit fly by quantitative RT-PCR. Afterwards, using different experimental techniques (RT-PCR of intermediate splicing products; RT-PCR test for lariat structures of intermediate introns; and mutational and deletion analyses of RP-sites) Burnette *et al.* [13] characterized in detail a mechanism that subdivides large introns by recursive splicing at non-exonic elements and alternative exons. The authors showed that RP-sites are 20-times more abundant in large Drosophila introns compared to their complementary strands as well as compared to their short introns.

In 2006 Grellscheid and Smith [14] showed that a pseudo-exon (a sequence within an intron flanked by bona fide 5′ and 3′ splice sites) in the rat tropomyosin gene was in fact most likely an alternative exon whose inclusion would lead to non-sense mediated decay. They also showed in their study on rat tropomyosin that a 5′ splice site followed the pseudo-exon 3′ splice site. They named this arrangement a “zero-length exon,” which is equivalent in its form to an RP-site. Thus, RP-sites may indeed exist in mammals as well, although, as the authors suggest in their discussion, the function of the zero-length exon in this particular case is not likely to be the same as the RP-sites used for the recursive splicing of long introns as in the studies of Burnette *et al.* [13] in Drosophila. Few other examples of RP-sites in mammals exist in the literature at this time. An alternative hypothesis of large mammalian intron splicing has been proposed but not tested in [15].

Since mammals as well as many non-mammalian vertebrates have many more large introns than Drosophila, it follows then that there ought to be some aid to the removal of large introns if these species do not rely upon recursive splicing. Thus, we have performed a large-scale bioinformatics analysis to understand the possible splicing mechanisms for large introns in various vertebrate species. In particular, we predicted the number of stem structures within large introns, hypothesizing that periodic hairpins with stable stems and large loops may be a possible mechanism for pre-mRNA folding which could aid splicing efficiency.

## Results

### Distribution of Large Introns

Table 1 gives the distribution of large introns (>50 kb) in thirteen completely sequenced genomes of both vertebrates and invertebrates. The genome sequencing quality varies significantly from species to species. This is reflected in the second to last column of Table 1, showing the percentage of unspecified nucleotides (non- A, T, C, or G) in the investigated large introns. Observe that some species (rat, cow, and sea urchin) have around 9% of their bases uncharacterized within large introns while other species (human, mouse, and fruit fly) have almost no uncharacterized bases. Even for the latter group of species there are different kinds of errors in the genomic databases, including sequencing, contig assembly, annotations, etc. (see the discussion in [16]). The reader should also note that some genes are still considered “hypothetical” and as such the counts in Table 1 are subject to future genome revisions.

**Table 1:** Large intron statistics and genome information by species.

Species	*# large introns (>50 kb)*	*Genome size (x10^9^ bp)*	*# introns per gene*	*large intron quality (% N's)*	*large intron fragment quality (% N's)*
Human	3473	3.4	9.37	0.001	0.006
Mouse	2435	3.2	9.35	0.247	0.004
Rat	2332	3	9.17	8.442	0.672
Cow	2245	3.6	8.21	7.900	0.049
Dog	2223	3.4	9.79	0.572	0.004
Opossum	3270	3.5	8.88	1.495	0.028
Chicken	853	1.2	10.33	1.614	0.288
Zebrafish	756	1.9	8.29	4.926	0.892
Sea urchin	209	0.9	6.86	18.73	2.187
Fruit fly	45	0.2	3.98	0.000	0.004
Mosquito	7	0.27	3.3	0.122	0.154
Bee	199	0.19	6.2	1.578	0.199
Beetle	100	0.21	5	8.277	0.613

Note. For columns left to right: number of non-redundant large introns (>50 kb) in different animal genomes; genome size of each species; number of introns per gene; sequence quality of all large introns in the species (>50 kb) as measured by percentage of ambiguous nucleotides (number of N's); sequence quality in the random set of large intron fragments used to predict stems.

### Distribution of Splicing Site Motifs inside Large Introns

**Figure 1:**
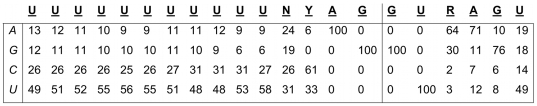
Ratcheting point consensus sequence (RP-site). The RP-site consensus was obtained from our purged sample of 11,315 non-redundant human gene sequences (with <50% sequence identities between each other) from the human Exon-Intron Database, release 35p1. The top row contains the consensus sequence derived from the frequency information below. Each nucleotide in the consensus sequence is a column in the matrix whose rows show the frequencies found for each given nucleotide at that position. The first column gives the nucleotides corresponding to the frequency information.

Table 2 shows the distribution of combined donor/acceptor sites for recursive splicing (RP-sites) within large introns and within their complementary strands. (For a study of RP-sites according to intron size class, see Supplementary Figure S1.) We scored RP-sites based on the human splice junction consensuses as shown in Figure 1. However, very similar results were obtained when we used the splice junction consensuses of fruit fly, chicken, or zebrafish. Table 2 demonstrates that all of the studied invertebrates had a considerable enrichment of RP-sites within their large introns in contrast to their complementary sequences, which were used as the control. Contrary to this observation, mammals and other vertebrates had a much smaller abundance of RP-sites within their large introns compared to their complementary strands (a ratio of 1.5 or less). Supplementary Figure S1 demonstrates that RP-sites are many times more abundant in the larger introns of Drosophila than in its shorter introns, but in humans, intron size does not affect this enrichment. Additionally, Supplementary Figure S2 graphs the RP-site, 5′- and 3′- splice site enrichment ratios for all species. We also detected that mammalian and other vertebrate large introns had more stringent splice site motifs at their termini (the average score of large intron splice sites exceeded the average score of medium-sized introns by 10%).

**Table 2 pone-0007853-t002:** Number of RP-sites per 100 kb inside large introns and their complementary sequences.

Species	Introns (>50 kb)	Complementary Strands	RATIO (intr/comp)
Human	0.122	0.082	**1.5**
Mouse	0.087	0.078	**1.1**
Rat	0.078	0.074	**1.0**
Cow	0.112	0.098	**1.1**
Dog	0.139	0.107	**1.3**
Opossum	0.135	0.108	**1.2**
Chicken	0.105	0.102	**1.0**
Zebrafish	0.112	0.120	**0.9**
Sea urchin	0.540	0.066	**8.2**
Fruit fly	2.196	0.101	**21.7**
Mosquito	1.029	0.000	***>>10***
Bee	0.484	0.122	**4.0**
Beetle	0.807	0.101	**8.0**

Note. The given ratio is the number of RP-sites of large introns to the number of RP-sites of the *complementary* sequences of the same large introns.

In a similar manner, we calculated the distribution of donor and acceptor splice site motifs within the same set of large introns and their complementary strands (Tables 3 and 4 respectively). These computations were also based on the human splice junction consensuses with the assumption that 5′- and 3′-intron termini (GT or AG dinucleotides respectively) must be present in the RP-site motifs. These sites were counted when their scores exceeded eighty percent. It is clear from Tables 3 and 4 that the large introns of all studied species do not have any extreme excess of donor or acceptor splice sites compared to their complementary strands. This result stands in contrast to Table 2 and serves as a baseline for how often we should expect to find RP-sites on the complementary strand.

**Table 3 pone-0007853-t003:** Number of donor splice sites per 100 kb inside large introns and inside the complementary sequences of the same large introns.

Species	Intron (>50 kb)	Complementary Strands
Human	34.102	35.257
Mouse	33.949	36.444
Rat	30.953	33.625
Cow	29.777	30.893
Dog	33.750	35.270
Opossum	37.091	39.522
Chicken	33.693	30.768
Zebrafish	24.940	25.740
*Sea urchin*	25.565	24.432
*Fruit fly*	19.836	22.235
*Mosquito*	20.792	24.292
*Bee*	14.722	17.225
*Beetle*	23.996	23.025

**Table 4:** Number of acceptor splice sites per 100 kb inside large introns and inside the complementary sequences of the same large introns.

Species	Introns (>50 kb)	Complementary Strands
Human	10.012	7.764
Mouse	4.770	3.672
Rat	3.877	2.905
Cow	4.442	3.165
Dog	9.348	7.039
Opossum	4.417	3.540
Chicken	6.250	4.836
Zebrafish	5.078	4.694
Sea urchin	2.945	2.483
Fruit fly	4.900	3.548
Mosquito	3.706	1.235
Bee	5.458	5.191
Beetle	3.062	1.840

As an additional control, we used intergenic regions from human and fruit fly (see Methods) and measured the RP-site frequencies in those regions. Enrichment ratios of RP-sites for large introns versus their complementary sequences in human and fruit fly are 1.5 and 27.5 respectively. However, when we use intergenic regions as the control frequency, the RP-site ratios of large introns to intergenic regions are 1.3 and 29.7 for human and fruit fly respectively (see Supplementary Figure S3).

### Searching for Double-Stranded Secondary-Structures inside Large Introns

**Figure 2:**
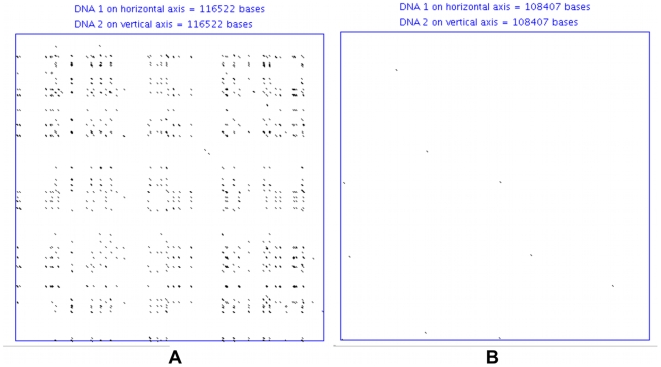
Human and Drosophila large intron dot-plots. (**A**) A dot-plot of human intron 21 from the *CNTNAP2* gene versus its complementary sequence. (**B**) Dot-plot of the drosophila intron 1 from the *luna* gene versus its complementary sequence. Here the dot-plot window size is 19 and the mismatch limit is set to 0. Low complexity repeats were filtered out using *RepeatMasker* before performing the dot-plot. The diagonal lines on the graph represent base pairing between different sections of the large introns that we may interpret as potential stem structures. The dot-plot conveys all possible combinations of stems in the sequence.

RNA hairpin structures are crucial for the splicing of group I and group II introns [23]–[24]. A correlation between secondary structure of pre-mRNA spliceosomal introns and the efficiency of splicing has been described [25]. Hairpins inside spliceosomal introns can also regulate alternative splicing in many eukaryotic genes [26]. These facts give us the motivation to examine the abundance of possible hairpin structures in the large introns of vertebrates and to understand the role they might play in efficient splicing. Indeed, since vertebrates do not show an abundance of RP-sites we suppose that they must have some other mechanism for efficiently splicing large introns, which might be intron folding via multiple sequential hairpin structures. One of the simplest ways to visualize such hairpin structures is a dot-plot comparison of an intron sequence against its complementary strand, which is shown in Figure 2. Sequence segments that could form possible stem structures are plotted as short diagonal lines in this figure. Typical dot-plots for human and fruit fly large introns are given in Figures 2A and 2B respectively (human intron 21 of the *CNTNAP2* gene versus its complement and drosophila intron 1 of the *luna* gene versus its complement). Using *RepeatMasker* in this dot-plot analysis, we excluded all simple and low-complexity repeats (micro-satellites, e.g. poly-AT sequences) from the analysis since they have an ability to interact with the nearest neighbor repetitive units rather than with more remote ones. In human, as with other mammals, the dot-plot detected a good number of matched segments throughout the entire large intron while the fruit fly showed very few possible stem structures. Examination of the predicted stem sequences of large introns showed that these possible stems are primarily formed by interspersed repeats belonging to the SINE and LINE classes. In the case of humans, the vast majority of the predicted stems are formed by any two oppositely oriented *Alu*-repeats, and, to a much lesser extent, *L1* or *L2* LINE repeats (see Table 5).

**Table 5:** The DNA repeats associated with the predicted stems of Drosophila and human large intron fragments.

A. *Drosophila*			
Count	Percent	Average Overlap *Length* (bp) of Stem Sequences with Repeat Family	Repeat Family
2	100%	31	Simple Repeat

B. *Human*			
Count	Percent	Average Overlap *Length* (bp) of Stem Sequences and Repeat Family	Repeat Family
1534	81.7%	149	SINE/Alu
160	8.5%	259	LINE/L1
69	3.7%	118	LTR/MaLR
58	3.1%	39	Simple Repeats
26	1.4%	*0*	*No Repeat Overlap*
31	1.9%	*N/A*	*All Other Repeats*

Note. The unique, predicted stems are from the same set of randomly selected large intron fragments from Table 6. Repeat families use *RepeatMasker* categories with the exception of “No Repeat Overlap”, implying no such overlap was found between the strands of the predicted stems and repetitive elements, and “All Other Repeats” which is used to aggregate all other repeats less frequent than the “No Repeat Overlap” category. The average length of stem-repeat overlap for each repeat family is also given.

The direct computational method for the prediction of secondary structures in long RNA sequences, such as large introns, is not feasible because of the enormous sequence length [27]. Therefore, we first gathered potential stable stem structures indirectly by using BLAST alignments (and the dot-plots for visual inspection) of large intron sequences versus their complementary strand. Next, we applied the RNAcofold program to this loose dataset to actually predict stem structures—retaining all unique stems with an MFE ≤−60 kcal/mol (see Table 6). We established that the actual choice of this threshold in the broad range of less than −50 to more than −100 kcal/mol has insignificant impact on the conclusions to the data. Thresholds higher than −50 kcal/mol represent much less stable structures. In the mammalian pre-mRNA sequences there are a number of local structures of this strength and it is highly questionable that stable hairpins with thousands of nucleotides long loops could exist. The analysis revealed that almost all stable stems were formed by interspersed DNA repetitive elements in vertebrates and by simple repeats (except in the beetle) in invertebrates. Further examination of interspersed repeats in human large introns revealed that the human *Alu* repeats distributed with the same frequency in the (+) or (−) orientations and were randomly positioned along the intronic sequence. In short, we were unable to detect any pattern in the location and orientation of the repetitive elements compared to models where we randomly placed such elements along introns. It is also interesting to note that *Alu* elements were more common in human intergenic regions than human large introns and that, if transcribed, the number of predicted stems in intergenic regions would also be larger.

**Table 6:** The features and frequencies of predicted stems for various species.

*Species*	*Number of 50 kb Intron Fragments*	*Stems per 50 kb*	*Average Stem Length (bp)*	*Avg. MFE of Stems (kcal/mol)*	*Average Loop Size (kb)*
Human	100	9.39	158	−258	12.3
Mouse	100	6.44	141	−229	13.5
Rat	100	5.54	156	−253	13.5
Cow	100	14.00	188	−310	14.4
Dog	100	8.02	112	−200	13.2
Opossum	100	5.73	138	−198	15.2
Chicken	100	1.36	95	−165	14.8
Zebrafish	100	8.72	114	−169	12.4
Sea Urchin	30	6.70	96	−142	10.8
Fruit Fly	30	0.03	32	−61	14.6
Mosquito	7	1.43	66	−114	12.6
Bee	30	0.53	56	−88	9.8
Beetle	30	4.00	155	−188	8.0

Note. Left to right we have the given species, the number of randomly selected large intron fragments (50 kilobases), the average number of stems per 50 kilobases, the average stem length, the average minimum free energy (MFE) of the stems, and the average loop size of the stems (in kilobases). All predicted stems were filtered to be less than or equal to −60 kcal/mol.

Our human intergenic region sample contained a total of 53.61% DNA repeats with 22.06% of the intergenic region being SINE compared to the sample of large introns which had 44.4% repeats with 12.36% SINE. For fruit fly, a 7.54% repeat composition in large introns jumped to 26.75% in intergenic regions (large retroviruses such as the ROO element appear in intergenic regions). Correspondingly, there were 19.56 unique, predicted stems per 50 kb in human intergenic regions versus 9.39 in human large introns (≤−60 MFE). Drosophila had 1.16 predicted stems per 50 kb in intergenic regions versus 0.03 in fruit fly large introns.

We detected negligible numbers of predicted stem structures formed by the ancient mammalian-wide interspersed repeats (MIR repeats from SINE class) presumably because they have accumulated too many mutations within each repetitive element to be adequately paired. Drosophila's only source of predicted stems came from simple repeats. See Table 5 for a full comparison of the human and drosophila DNA repeats that were associated with their respective predicted stems.

It is interesting to observe the sheer difference in magnitude in the number of stems between human and fruit fly. The average number of unique, predicted stem structures per 50 kb of large introns in different species is presented in Table 6. (We use the term “unique stem structures” to mean that any of the predicted stem's strands do not overlap with any other stem's sequences nor with each other.) Table 6 shows that these stems are about 1.4 to 420 times more abundant in mammalian large introns than in insects. The average lengths of these predicted stems are also given in Table 6, which shows that vertebrates only have at most 5.9 times the length of the stems found in invertebrates. However, from Table 6 it may also be argued that there is a trend for more stable stem structures in mammals and other vertebrates than in most invertebrates.

**Figure 3:**
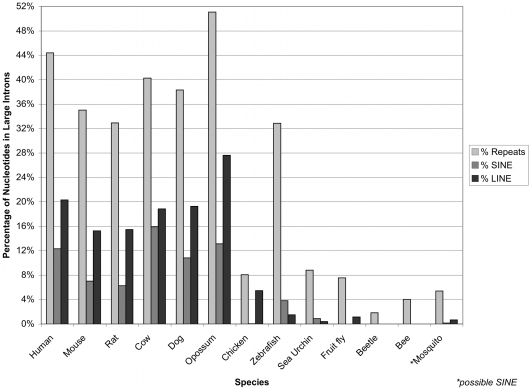
Repetitive elements within species. The percentage of repeats for the complete set of large introns for various species. The light gray bars are for the total percentage of repeats in large introns (percentage of nucleotides), the medium gray bars are only for the percentage of nucleotides made up by short interspersed element (SINE) repeats, while the dark gray bars are only for long interspersed element (LINE) repeats. *Note: Mosquito contains an ambiguous SINE element called “SINEX-1_AG”.

**Figure 4:**
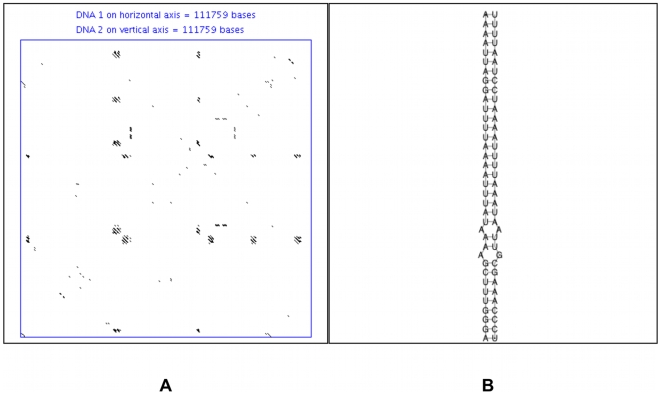
Beetle large intron dot-plot and secondary structure. (**A**) Dot-plot of a beetle large second intron of the predicted gene XP_968205.1. The window size for the dot-plot was 19 and the mismatch limit was 0. (**B**) An example stem from the same intron, created using RNAcofold (it is not associated with any known repeat).

Apart from stems, we studied the composition of repetitive elements within large introns using the *RepeatMasker* program. The results are presented in Figure 3 and demonstrate that all of the studied invertebrates have no or negligible amounts of short or long interspersed repetitive elements, while mammals and non-mammalian vertebrates have the highest representation of these types of repeats. Of interest, the red-flour beetle (*Tribolium castaneum*) has the highest number of unique stems predicted for any insect as well as the most stable predicted stem structures for all insects. (Sea urchin, the only other invertebrate, has more predicted stems; however, they are primarily associated with simple repeats, see Discussion.) Strangely though, Figure 3 shows that beetle large introns have fewer repetitive elements than the rest of the studied invertebrate species. An example dot-plot for an entire beetle large intron is shown in Figure 4A while one example stem from the beetle stem prediction is shown in Figure 4B. One may conclude that beetle large introns do possess a potential for stem structures that is unique among the studied insects, although these structures typically are not quite as abundant as the stems predicted for mammalian large introns. The repeat composition for the predicted stems of beetle large intron fragments (data not shown) reveals that over 90% of the stems are not associated with any known repeats.

## Materials and Methods

The sequences of non-redundant, large introns (>50 kb) were obtained from the Exon-Intron Database [16]. Our datasets are available upon request. Supplementary Figure S1, Figure 3, and Tables 1–4 used these datasets. For the human intergenic region RP-site analysis we used the same data set as in [17], which contains over 3.5 million nucleotides. For the fruit fly intergenic region RP-site analysis we used the complete set of intergenic regions from FlyBase release 5.10 (ftp://ftp.flybase.net/genomes/Drosophila_melanogaster/) [18]. FlyBase release 5.10 was the same release used to build our Exon-Intron database from which we obtained the sample of large introns. See Supplementary Figure S3 for intergenic region RP-site analysis.

For the recognition of RP-sites we used the same computational algorithms as published by Burnette *et al.* 2005 with the same 80% scoring threshold for counting the number of RP-sites. In this computation we assumed that all RP-sites must have an invariable core sequence of AG|GT representing the intron's dinucleotide termini. The consensus for intron splicing junctions was obtained from our purged sample of 11,315 non-redundant human gene sequences (with <50% sequence identities between each other) from the human Exon-Intron Database, release 35p1 [16]. Additionally, when comparing Drosophila with human introns in Supplementary Figure S1 we used the Drosophila consensus matrix to detect RP-sites in fruit fly and the human consensus matrix to detect RP-sites in *Homo sapiens*. Various scoring thresholds for human and Drosophila were used: 80%, 70%, and 60% with 80% being the highest quality RP-site recognition threshold. The intron size classes chosen for this analysis (1–6 kb, 6–17 kb, 17–41 kb, 41–100 kb, and 100+ kb) had a total of between 203 and 212 million bases for human. The fruit fly intron size classes were held to the same intervals allowing direct comparison of intron class size between Drosophila and human. For human and fruit fly RP-site analysis in intergenic regions we used the respective human and fruit fly consensus matrices.

The data used in the stem prediction and analysis (see Tables 5–6) was a randomly extracted set of large intron fragments from the datasets used in the RP-site analysis. We extracted 50,000 bp of fixed sequence fragments randomly from each of the large intron datasets of each species, but taking no more than one fragment from any particular large intron. For invertebrates (except mosquito), we randomly selected 45 fragments of 50 kb each and kept the 30 highest quality (by lowest number of N's) fragments. For mosquito, we randomly extracted 50 kb sequence fragments from each sequence in the mosquito large intron dataset (7 large intron fragments). For each vertebrate species we randomly selected 150 large intron fragments of 50 kb each and kept the 100 highest quality fragments. With respect to intergenic regions in human and fruit fly, we randomly extracted 100 fragments of 50 kb a piece from the respective datasets. The intergenic region fragments for human and fruit fly contained no ambiguous nucleotides. For a summary of the fragment quality of large introns, please see the last column of Table 1.

For the stem prediction, we initially gathered a rough pool of possible stems using blast2 alignments of large introns versus their complementary sequences. We used default parameters for *blastn* and matched only to the top or forward strand. From the blast2 alignments we actually predicted the stems using the RNAcofold program (Vienna package 1.6.1) with default parameters [19]. A custom perl program was used in concert with RNAcofold to: (a) retain structures with a minimum free energy (MFE) less than or equal to −60 kcal/mol; (b) discard palindromic structures (stems with no loops); (c) retain only unique stems; and (d) calculate statistics such as the average MFE, average stem length, and average loop size of the predicted stems. Predicted stems were considered *unique* if the stem's strands did not overlap with any other stem's sequences in the predicted stem set. Moreover, if a new stem to be added overlapped only one stem in the set and if the new stem had an MFE within 10% of the old stem and a smaller loop size, the algorithm would replace the old stem with the new one. The results are presented in Table 6.

Masking and characterization of repetitive elements inside introns (and intergenic regions) were performed with *RepeatMasker Open-3.1.8* (www.repeatmasker.org) using the sensitive/slow search mode and species/genus specific repeat libraries [20]. The repeat libraries used by *RepeatMasker* was database release *20061006* with *WUBlast 2.0MP* (http://blast.wustl.edu) to perform the scanning [21].

Cross-referencing between predicted stems sequences and RepeatMasker data for the large intron fragments of human and drosophila was performed using a custom perl program. The coordinates of the two sequences forming a predicted stem were each individually cross-referenced against the locations of all repeats in the respective 50 kb large intron fragment. For Table 5, if any overlap were found between the predicted stem sequences and a repeat it was counted. However, in order to verify the strength of the association between the repeats and the predicted stems, the lengths of their overlap for each repeat family was kept and the average length of the sequence overlap was calculated. The “No Repeat Overlap” category in Table 5 is not a RepeatMasker repeat family and has a 0 nucleotide average overlap length by definition. The “All Other Repeats” category in Table 5 are the aggregate count all other repeat families whose count is less than the “No Repeat Overlap” category. The average length of overlap is omitted for the “All Other Repeats” category since it contains many different repeat families each of a very low occurrence whose average is not reliably interpretable.

Dot-plot analysis was performed using a modified version of the Java applet “Nucleic Acid Dot Plot” [22]. The parameters used for Figures 2 and 4 included a window size of 19, a mismatch limit of 0, and masking of low-complexity repeats as X's.

All other computations were performed by programs written in perl and with queries performed in MySQL—all available upon request.

## Discussion

The timely removal of large introns from pre-mRNA poses a challenging problem to spliceosomal machinery. It has been experimentally and computationally proven that in *Drosophila melanogaster* there exists a special strategy named recursive splicing for the excision of large introns. Recursive splicing occurs via selective accumulation of combined donor-acceptor splicing sites called RP-sites [12]–[13]. In our research, we used the complete set of Drosophila large introns to confirm the previous computation by Burnette *et al.* [13]—showing again that fruit fly had more than 20 times the selective accumulation of RP-sites within large introns over their complementary strands. Similarly, all other studied Insecta species (mosquito, honey bee, and beetle) as well as more distant invertebrates (sea urchin) also had an accumulation of RP-sites within their large introns that was several times more abundant when compared to their complementary strand. We also showed that the accumulation of RP-sites is in particular with respect to intron class size in fruit fly but not in human (Supplementary Figure S1). On the other hand, all studied vertebrates, including six mammals, did not show significant accumulation of RP-sites (see Supplementary Figure S2 for a visual representation of this phenomena). Moreover, vertebrate species have overwhelmingly more large introns than the examined invertebrates. Therefore, vertebrates must mobilize another molecular mechanism for the removal of their large introns from pre-mRNA. We have hypothesized that multiple hairpins with large loops could form compact spatial structures within large introns that could help put the donor and acceptor splice sites in close proximity in order to facilitate splicing.

To test this conjecture, we examined the distribution of possible stable stem structures inside the large introns of vertebrates and invertebrates. It appeared that within Drosophila's large introns, stem structures are practically absent. The same trend was observed for the invertebrates honey bee and mosquito. On the other hand, in mammals, multiple SINE and LINE repeats (primarily SINE) located in different orientations throughout large introns drive the potential formation of hairpins with large loops. For humans there were an average of about 9.4 possible hairpins per 50 kb of the analyzed large intron sequence fragments. A vast majority of these possible stems are formed by oppositely oriented primate-specific *Alu-*repeats (81.7%). Other investigated mammals do not have *Alu-*elements, but other types of evolutionarily new SINEs specific for their taxa. These SINEs could also allow for the formation of multiple hairpin structures inside large introns. Only one of the studied vertebrates, chicken, does not have SINE elements in its genome. Instead, the chicken has very abundant and relatively short LINE elements that comprise over 60% of its repetitive elements. Thus in chicken large introns, possible stems may be formed solely by LINE repeats and not SINE repeats. One may observe, however, that the chicken has very few predicted stems, less than all studied vertebrate species and comparable to some insect species. It may be the fact that avian genomes deal with large intron splicing differently than other vertebrate species. Two facts though are clear: predicted stems for chicken are quite strong and stable (see Table 6) and the chicken has several times fewer large introns than all studied mammalian species (see Table 1).

Interestingly, the beetle and especially the sea urchin contain the most predicted stems of all studied invertebrates. While the sea urchin may contain the most predicted stems, even comparable to zebrafish, the majority of these predicted stems (47.5%) overlap with simple and low complexity repeats that might form hairpin structures without loops instead of the stems with large loops that we predict in mammals. Curiously, beetle's predicted stems are not strongly associated with any particular kind of repeat. We suppose that the beetle predicted stems might be formed by as yet unidentified repeats, or that they are merely a part of more complicated RNA secondary structures.

The average number of predicted long and stable stems in large introns of different mammals is 5.5 to 14 per 50 kb of large introns (see Table 6). These stems create large loops with the average size of 12.3 to 15.2 kilobases. Relatively large loops with lengths up to 3 kilobases are characteristic for group I and group II introns containing ORFs. According to [28], about 30% of group I introns and about 25% of group II introns code proteins. These coding sequences are located inside loops that do not have specific secondary structures. The ORF-containing loops of group I introns are around 1000 nucleotides in length, while those of group II introns are even larger. The latter code proteins with an average size of 500–600 aa, according to the Group II intron database [29]. Moreover, some of these proteins are significantly larger (up to 1064 aa in M.p.atpAI1 intron [29]). Interestingly, these large ORF-containing loops of group I and II introns have relatively short terminal stems, usually no longer than 12 nucleotides with MFE weaker than −10 kcal/mol (P6 or P8 stems for group I; IV stems for group II introns). Multiple hairpins of these introns form complex 3D structures. These complex 3D-structures include pseudoknots and non-Watson-Crick base pairing. Presently, there are no reliable algorithms/programs to properly calculate the free energy of such structures. Therefore we do not provide such estimations. However, each individual stem of group I and II introns has folding energy at least ten times weaker than −258 kcal/mol–the average minimum free energy of the predicted stems of large introns in human (see Table 6). Therefore, it is reasonable to hypothesize that numerous SINE and LINE repetitive elements within large mammalian introns are able to form multiple large hairpins with 100–300 nucleotide-long stems and up to a 15 kb long loops. Such structures might help to bring donor and acceptor splicing junctions of large introns closer to each other, and, thus, facilitate the effectiveness of their splicing. Indeed, recently it has been shown that even in the short introns of Saccharomyces cerevisiae secondary structures facilitate splicing by bringing together splicing elements [25].

Insertion of interspersed retrotransposon elements, such SINEs and LINEs, is a major force for the expansion of the genome size as a whole and intron sizes in particular [30]. Accumulation of new types of retrotransposons occurs gradually and could take millions of years. After gaining several interspersed repetitive elements inserted in opposite orientations inside an intron, these elements could allow for the formation of hairpin structures with long stems to be formed by the base-pairing repetitive sequences. These hairpins would introduce a new spatial organization into intronic RNA by keeping donor and acceptor splice sites in close proximity. Such a spatial organization could become a novel mechanism for facilitating the splicing of large introns. If RP-sites were indeed already present, this competing mechanism for efficient splicing could, in turn, ease the selective constraints that preserve recursive splicing and decrease RP-site frequency to a random expectation. We therefore hypothesize that oppositely oriented interspersed repetitive elements may be playing this role in the large introns of vertebrate species. It is indeed interesting to consider that the possible problems caused by the expansion of introns due to the insertion of repetitive elements may at once be remediated by the base-pairing of the self-same elements. However, whatever forces drove or allowed the formation of such possible stem structures, their potential role in the efficient splicing of large introns poses an appealing question to molecular biologists, a question that is suggestive for future work *in vitro*.

## Supporting Information

Supplementary Figure S1 RP-site enrichment with respect to intron size. Human and Drosophila RP-site enrichment ratio calculated for various scoring thresholds and intron size classes. The RP-site ratio is the count of RP-sites on the direct strand of introns divided by the count of RP-sites on the complementary strand of said introns. Thresholds for scoring or recognizing RP-sites to a consensus sequence are 80%, 70%, and 60% with 80% being the most stringent (good quality) score. Intron class sizes are the five sets with individual intron lengths: 1) 1–6 kb, 2) 6–17 kb; 3) 17–41 kb; 4) 41–100 kb; and 5) larger than 100 kb. Note: Drosophila large intron group 100+ kb with scoring threshold 80% ratio is estimated, since 8 to 0 cannot be divided, using a polynomial curve fit (Rˆ2  = 1) to the previous four points.
(0.04 MB DOC)

Supplementary Figure S2 RP-site ratio comparison. In various species, the ratios of the number of sites (RP-site, 5 prime, or 3 prime) on the sense strand of large introns (>50 kb) is compared to the number of sites on the anti-sense strand of large introns.
(0.04 MB PDF)

Supplementary Figure S3 Comparison of controls used to calculate RP-site enrichment ratios in large introns. The complementary strand of the large introns is used as the first control. The RP-site enrichment ratio is the frequency of RP-sites in the direct strand of large introns to the frequency of RP-sites in the direct strand of large introns. A set of 50 kb intergenic region fragments is used as a second control. The RP-site enrichment ratio here is the frequency of RP-sites in the direct strand of large introns to the frequency of RP-sites in intergenic regions. Both human and fruit fly species are considered with RP-sites being calculated at the 80% scoring threshold. The human consensus matrix was used for human and the fruit fly consensus matrix was used for fruit fly. All frequencies are per 100 kilobases.
(0.03 MB DOC)
