# Supplementary material for: The Peculiarities of Large Intron Splicing in Animals
Source: PLoS One. 2009 Nov 16;4(11):e7853. doi: 10.1371/journal.pone.0007853 (PMC2773006; doi:10.1371/journal.pone.0007853)
Supplement: Supplementary Figure S1 — RP-site enrichment with respect to intron size. Human and Drosophila RP-site enrichment ratio calculated for various scoring thresholds and intron size classes. The RP-site ratio is the count of RP-sites on the direct strand of introns divided by the count of RP-sites on the complementary strand of said introns. Thresholds for scoring or recognizing RP-sites to a consensus sequence are 80%, 70%, and 60% with 80% being the most stringent (good quality) score. Intron class sizes are the five sets with individual intron lengths: 1) 1–6 kb, 2) 6–17 kb; 3) 17–41 kb; 4) 41–100 kb; and 5) larger than 100 kb. Note: Drosophila large intron group 100+ kb with scoring threshold 80% ratio is estimated, since 8 to 0 cannot be divided, using a polynomial curve fit (Rˆ2 = 1) to the previous four points. (0.04 MB DOC) [file pone.0007853.s001.doc]

| INTRON  SIZE CLASS | **RP-site Enrichment in Intron Size Classes by Species**  **and Scoring Threshold** | | | | | |
| --- | --- | --- | --- | --- | --- | --- |
|  | Human | | | Fruit Fly | | |
|  | *≥ 80% score* | *70%* | *60%* | *80%* | *70%* | *60%* |
| *1 to 6 kb* | 1.1 | 1.2 | 1.2 | 0.0 | 0.2 | 0.3 |
| *6 to 17 kb* | 1.4 | 1.3 | 1.3 | 2.5 | 1.2 | 0.8 |
| *17 to 41 kb* | 1.3 | 1.4 | 1.4 | 14.5 | 6.9 | 3.0 |
| *41 to 100 kb* | 1.6 | 1.3 | 1.3 | 36.0 | 10.0 | 6.1 |
| *100+ kb* | 1.5 | 1.3 | 1.3 | *67 | 12.0 | 5.3 |
| **estimated* | | | | | | |
