# Supplementary material for: The Peculiarities of Large Intron Splicing in Animals
Source: PLoS One. 2009 Nov 16;4(11):e7853. doi: 10.1371/journal.pone.0007853 (PMC2773006; doi:10.1371/journal.pone.0007853)
Supplement: Supplementary Figure S2 — RP-site ratio comparison. In various species, the ratios of the number of sites (RP-site, 5 prime, or 3 prime) on the sense strand of large introns (>50 kb) is compared to the number of sites on the anti-sense strand of large introns. (0.04 MB PDF) [file pone.0007853.s002.pdf]

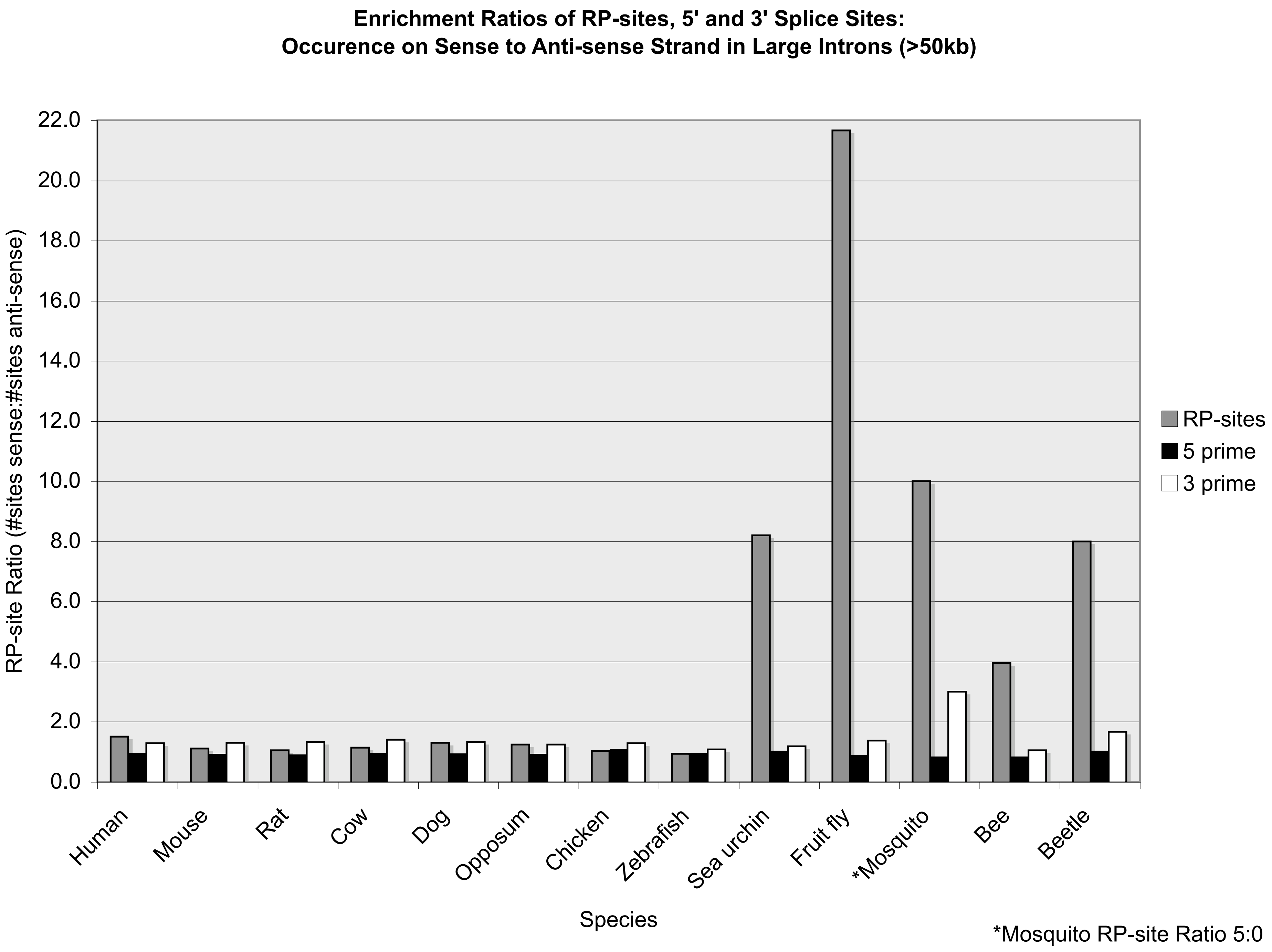
Enrichment Ratios of RP-sites, 5' and 3' Splice Sites:
Occurence on Sense to Anti-sense Strand in Large Introns (>50kb)
RP-site Ratio (#sites sense:#sites anti-sense)
22.0
20.0
18.0
16.0
14.0
12.0
10.0
8.0
6.0
4.0
2.0
0.0
Human
Mouse
Rat
Cow
Dog
Opposum
Chicken
Zebrafish
Sea urchin
Fruit fly
*Mosquito
Bee
Beetle
Species
RP-sites
5 prime
3 prime
*Mosquito RP-site Ratio 5:0
