# Supplementary material for: The Peculiarities of Large Intron Splicing in Animals
Source: PLoS One. 2009 Nov 16;4(11):e7853. doi: 10.1371/journal.pone.0007853 (PMC2773006; doi:10.1371/journal.pone.0007853)
Supplement: Supplementary Figure S3 — Comparison of controls used to calculate RP-site enrichment ratios in large introns. The complementary strand of the large introns is used as the first control. The RP-site enrichment ratio is the frequency of RP-sites in the direct strand of large introns to the frequency of RP-sites in the direct strand of large introns. A set of 50 kb intergenic region fragments is used as a second control. The RP-site enrichment ratio here is the frequency of RP-sites in the direct strand of large introns to the frequency of RP-sites in intergenic regions. Both human and fruit fly species are considered with RP-sites being calculated at the 80% scoring threshold. The human consensus matrix was used for human and the fruit fly consensus matrix was used for fruit fly. All frequencies are per 100 kilobases. (0.03 MB DOC) [file pone.0007853.s003.doc]

| **COMPLEMENTARY STRAND OF LARGE INTRONS AS CONTROL** | | | |
| --- | --- | --- | --- |
|  | *RP-sites per 100kb (Large Introns)* | *RP-sites per 100kb (Complementary Strand)* | *RP-site Enrichment Ratio* |
| *Human* | 0.122 | **0.082** | **1.5** |
| *Fruit Fly* | 1.859 | **0.068** | **27.5** |
| **INTERGENIC REGION AS CONTROL** | | | |
|  | *RP-sites per 100kb (Large Introns)* | *RP-sites per 100kb (Intergenic Region****)*** | *RP-site Enrichment Ratio* |
| *Human* | 0.122 | **0.096** | **1.3** |
| *Fruit Fly* | 1.859 | **0.063** | **29.7** |
